# Metachronous metastasis of renal cell carcinoma to the contralateral ureter 8 years after nephrectomy presenting as hydronephrosis

**DOI:** 10.1016/j.eucr.2026.103447

**Published:** 2026-04-15

**Authors:** Kolos K. Nagy, Caroline J. Cushman, Jason R. Jesudass, Seshadri D. Thirumala, Brian E. Nicholson, Donald P. Quick

**Affiliations:** a School of Medicine, Texas Tech University Health Sciences Center, Lubbock, TX, USA; b Department of Pathology, Covenant Medical Center, Lubbock, TX, USA; c Department of Urology, Covenant Medical Center, Lubbock, TX, USA; d Department of Hematology and Oncology, Covenant Medical Center, Lubbock, TX, USA

**Keywords:** Metachronous metastasis, Renal cell carcinoma, Hydronephrosis, Benign prostatic hyperplasia

## Abstract

Renal cell carcinoma (RCC) is characterized by a potential for unpredictable metastatic pattern and a capacity for delayed recurrence years after apparently curative surgical resection. Metastatic spread most commonly involves the lungs, bone, and lymph nodes, and involvement of the contralateral ureter is exceptionally rare, particularly when occurring as a true metachronous lesion years after primary tumor resection**.** Herein, we report a case of isolated contralateral distal ureteral metastasis identified 8 years after radical nephrectomy for RCC.

## 1 Introduction

Renal cell carcinoma (RCC) is the most common primary malignancy of the kidney, accounting for over 90% of kidney and 3% of all adult malignancies.^1^ Despite advances in surgical and medical management, RCC remains an aggressive neoplasm with a historically reported disease-specific mortality of approximately 30 - 40%.^2^

Following complete surgical resection, disease recurrence remains clinically significant, with approximately 20 - 30% of patients overall, and up to 40% of those with higher-risk localized disease, developing local recurrence or metastasis following nephrectomy. Up to one third of RCC recurrences occur more than five years after nephrectomy, with rare cases reported beyond 10 years, reflecting the tumor's capacity for prolonged dormancy and unpredictable metastatic behavior.^3^

Metastatic spread of RCC most commonly occurs via hematogenous routes through the renal vein and inferior vena cava. The most common sites of metastasis include the lungs (∼71%), lymph nodes (∼38%), bone (∼31%), liver (∼13%), adrenal glands (∼10%), and brain (∼8%).^4^ Less commonly, RCC may disseminate through the genitourinary tract. Tumor involvement of the ureter or bladder may occur through direct contiguous extension, microscopic intraluminal seeding, or implantation of detached tumor fragments, also known as “drop metastasis.” Drop metastases and microscopic tumor seeding most often involve the ipsilateral ureter and bladder wall, but contralateral involvement is uncommon, and presentation of metastatic lesions after excision of the primary tumor is exceedingly rare.^5^

The appearance of a metastatic lesion after a temporal interval following treatment of the primary tumor, rather than at initial diagnosis, is defined as metachronous metastasis.^6^ Isolated metachronous metastasis of RCC to the contralateral ureter years after nephrectomy is exceptionally uncommon, with only a limited number of cases reported in the literature. Such presentations can pose diagnostic and therapeutic challenges, particularly when there might be symptom overlap with benign urologic conditions.^3^^,^^6^

Herein, we report a rare case of metachronous RCC metastasis to the contralateral distal ureter incidentally discovered 8 years after radical nephrectomy, including a 6-year disease-free interval followed by delayed detection approximately 2 years after initial presentation, initially presenting as hydronephrosis in a patient undergoing evaluation for benign prostatic hyperplasia (BPH). This case highlights the importance of maintaining a high index of suspicion for late and atypical metastatic disease in patients with a remote history of RCC and demonstrates the need for long-term surveillance even after apparently curative resection of the tumor.

## 2 Case presentation

A 79-year-old male with a BMI of 30 kg/m^2^ presented to the clinic for BPH and urinary incontinence. Past medical history was significant for radical left nephrectomy for RCC 6 years prior, which was graded 4/4 T1b with negative surgical margins. The urogram obtained 4 months earlier and was unremarkable.

**Fig. 1:**
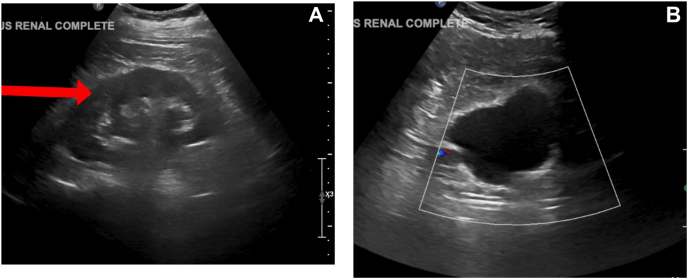
(A) Renal ultrasound long-axis view at initial presentation demonstrating mild to moderate hydronephrosis of the right kidney. (B) Bladder ultrasound transverse view at initial presentation demonstrating normal bladder morphology without visible obstruction.

Renal and bladder ultrasound (US) were ordered and demonstrated mild right hydronephrosis (Fig. 1a) and normal bladder with no visible obstruction (Fig. 1b). Renal function panel demonstrated elevated potassium at 5.3 mmol/L (ref: 3.5 - 5.1), elevated creatinine at 1.40 mg/dL (ref: 0.60 - 1.30), and decreased eGFR at 51 mL/min/1.73^2^ (ref: ≥60). Urinalysis revealed red, turbid urine with urine protein at 100 mg/dL (ref: 0 - 14 mg/dL), 3.5 urine red blood cells per HPF (ref: 0 – 2), and 0 - 5 hyaline casts per LPF (ref: 0 - 5).

One month later, the patient underwent aquablation for BPH, during which intraoperative cystoscopy revealed a patulous right ureteral orifice, suggesting this as a potential culprit in the patient's hydronephrosis. The patient tolerated the procedure well, and reported improvement in his symptoms in the immediate follow up.

**Fig. 2:**
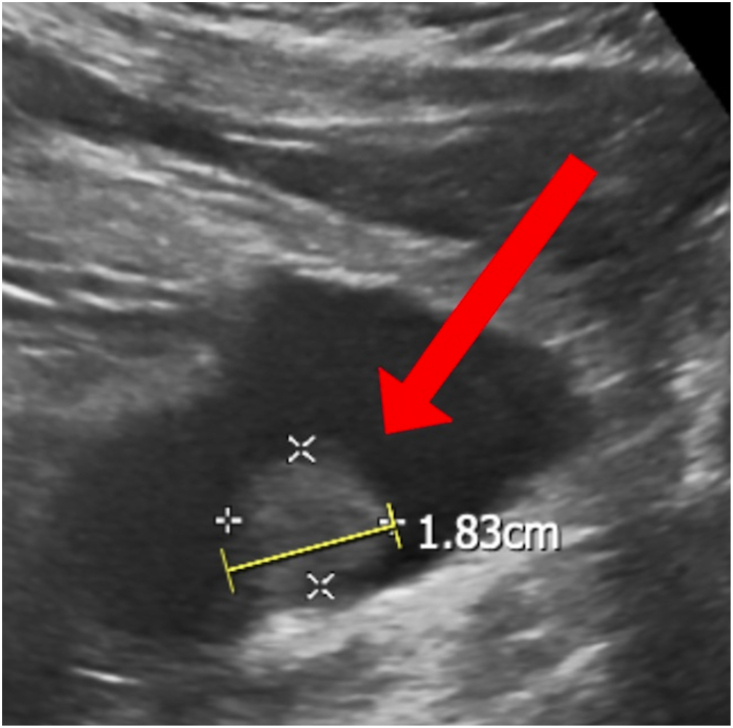
Bladder ultrasound transverse view obtained two years after prostate aquablation demonstrating a new mass along the right lateral bladder wall measuring approximately 1.8 cm in maximal diameter.

Two years after aquablation, the patient re-presented for worsening urinary retention, overflow incontinence, and gross hematuria. A bladder US was ordered, which demonstrated a mass on right bladder wall measuring 1.83 cm in diameter (Fig. 2).

**Fig. 3:**
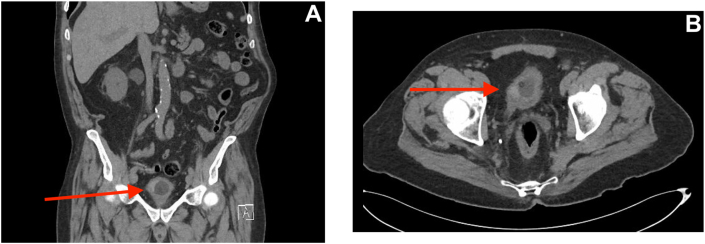
(A) Coronal non-contrast CT of the abdomen and pelvis demonstrating moderate dilation of the right collecting system and ureter with an obstructing lesion at the level of the right ureteral insertion and no mass or adenopathy at the site of prior left nephrectomy. (B) Axial non-contrast CT of the abdomen and pelvis demonstrating a mucosal-based obstructing lesion at the right ureteral orifice with associated upstream ureteral dilation.

Non-contrast CT of the abdomen and pelvis demonstrated moderate dilation of the right collecting system and ureter without evidence of calculi, along with diffuse bladder wall thickening and a mucosal-based obstructing lesion at the right ureteral insertion. The right kidney showed no abnormality or radiographic evidence of malignancy. No left kidney was identified and there was no mass or adenopathy identified at site of prior left nephrectomy (Fig. 3a and b).

A cystoscopy guided transurethral resection revealed a 2 cm tumor on a thin stalk coming from the right ureteral orifice. The patient's bladder had 2+ trabeculations without cellules, calculi, or foreign bodies. The tumor was resected via rigid biopsy forceps.

**Fig. 4:**
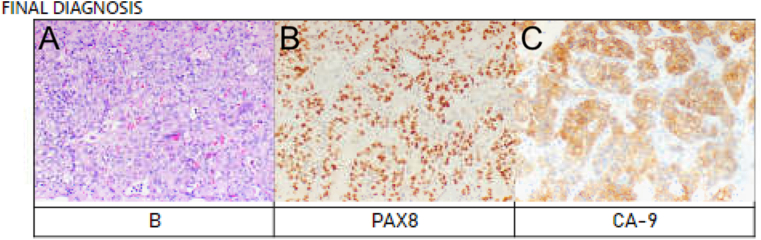
(A) Histopathologic section of bladder biopsy demonstrating papillary cystitis without evidence of dysplasia or malignancy. (B) Immunohistochemical staining of the ureteral tumor demonstrating strong nuclear positivity for PAX8. (C) Immunohistochemical staining of the ureteral tumor demonstrating diffuse positivity for carbonic anhydrase IX (CA-IX).

The biopsy of the bladder mass revealed erosions with papillary cystitis with no evidence of dysplasia or malignancy. Sections from the right ureteral tumor demonstrated tissue arranged in large groups and sheets with packeting of cells. The tumor cells were diffusely and strongly positive for renal markers including PAX8 and CA–9, while negative with all other antibodies tested, including GATA3, CK7, p63, and NKX3.1, excluding urothelial and prostate cancers. The tumor cells were positive for CK and negative for chromogranin and synaptophysin, ruling out paraganglioma of the bladder (Fig. 4a, b, 4c). The findings were consistent with metastatic clear cell renal cell carcinoma, identical with the histology of the primary tumor.

Three months after the resection, right ureteroscopy found obvious tumor recurrence a few centimeters proximal to the ureteral orifice. A right distal ureterectomy with ureteroneocystostomy was performed, and the small residual tumor was resected with negative margins. The pathological evaluation confirmed RCC recurrence with negative surgical margins, low-grade histology. There was no radiographic evidence of residual or metastatic disease. The ureteral stent was removed one month following resection, and subsequent US demonstrated no evidence of right hydronephrosis. Systemic therapy was not initiated, and the patient was managed with surveillance cystoscopy at 6-month intervals and annual abdominal imaging, with no recurrence identified to date. Taken together, these findings indicate an approximately 8-year interval between nephrectomy and diagnosis of contralateral ureteral metastasis.

## 3 Discussion

RCC is characterized by an unpredictable metastatic pattern and a unique ability to recur years after apparent disease eradication. While metastatic involvement of common organs such as the lungs and bone is well documented, metastasis to the contralateral ureter remains exceptionally rare. Among a limited number of reported cases of contralateral ureteral metastasis from RCC, fewer than 15 have demonstrated a true metachronous presentation according to Bretterbauer et al. (Table 1).^7^ The 8-year interval observed in this patient is longer than that reported in most previously published cases of contralateral ureteral metastasis from RCC, emphasizing the uniquely delayed and indolent nature of this recurrence.^5^^,^^7^ Recognition of this rare metastatic pattern is clinically relevant, as delayed ureteral metastases may masquerade as benign obstructive urologic disease, leading to diagnostic delay in patients with a remote history of RCC.

**Table 1:** Reported cases of metachronous renal cell carcinoma metastasis to the contralateral ureter following radical nephrectomy, including time to recurrence, patient demographics, clinical presentation, year of publication, and primary author.

Time between radical nephrectomy and diagnosis of contralateral ureteral metastasis	Patient age and sex	Presentation	Year of publication	Author(s)
5 months	61 yoM	hydronephrosis	2015	Dixon et al.^8^
14 months	62 yoM	hematuria	2006	Zorn et al.^9^
4 years	74 yoM	hematuria	2011	Zhang et al.^6^
7 years	71 yoM	hematuria	2017	Bretterbauer et al.^7^
7 years	70 yoM	hematuria	2021	Katsui et al.^10^
6 years	79 yoM	Urinary retention & hydronephrosis	2025	Case reported here

The prolonged interval between apparent complete surgical excision and detection of metachronous metastasis suggests early microscopic tumor seeding with prolonged dormancy, a mechanism consistent with the known biology of RCC, which may remain clinically silent for extended periods before manifesting as metastatic disease.^11^ The contralateral location of the ureteral lesion, absence of ipsilateral renal recurrence, and its isolated nature argue against direct extension or synchronous metastatic spread and instead support microscopic intraluminal tumor cell seeding with prolonged dormancy**,** rather than classic drop metastasis or direct extension, as the most plausible mechanism.^6^ Interestingly, the metastatic lesion demonstrated lower-grade histology than the primary tumor. This finding may reflect intratumoral heterogeneity, clonal selection, or temporal evolution of tumor biology, all of which are well described in RCC and may contribute to variability in metastatic behavior.^6^^,^^11^

The diagnostic evaluation in this case was challenging, as the patient initially presented with symptoms attributable to BPH and hydronephrosis with no clinical suspicion of RCC. It remains unclear whether the patient's initial hydronephrosis was attributable to an early, radiographically occult ureteral lesion or to BPH. The discovery of a bladder-adjacent mass raised concern for primary urothelial malignancy, which accounts for the vast majority (>90%) of tumors arising in the bladder and upper genitourinary tract.^12^

As with other atypical presentations of RCC, management of isolated metachronous metastasis remains individualized. In this patient, given the absence of radiographically identifiable evidence of metastatic disease, complete resection of the lesion, low risk histology, and negative surgical margins, systemic therapy was deferred in favor of active surveillance.^13^^,^^14^

The patient's initial tumor was a high-grade WHO/ISUP grade 4 (Fuhrman 4/4 equivalent) pT1b RCC with negative surgical margins, for which the NCCN recommends risk-adapted surveillance following nephrectomy, including baseline abdominal CT, MRI, or ultrasound 3 - 12 months postoperatively and annual abdominal imaging thereafter, with annual chest radiography for three years. Current NCCN Clinical Practice Guidelines in Oncology for Kidney Cancer emphasize that post-nephrectomy surveillance should be tailored according to tumor- and patient-specific risk factors, while systemic therapy is generally reserved for metastatic disease not amenable to complete surgical resection, high-volume or rapidly progressive recurrence, synchronous metastatic presentation, unfavorable histologic features (e.g., high-grade disease, rhabdoid features, or sarcomatoid differentiation), or advanced stage or grade tumors (e.g., WHO/ISUP grade 3 - 4 or ≥ pT3 disease).^1^^,^^14^

This case further highlights the importance of multidisciplinary collaboration in managing atypical RCC presentations where urologic evaluation facilitated timely surgical intervention, pathologic interpretation established the diagnosis through immunohistochemical confirmation, and oncologic input guided postoperative management and surveillance strategy.

## 4 Conclusion

This case demonstrates the aggressive and unpredictable metastatic potential of renal cell carcinoma (RCC) and the necessity for continued vigilance following nephrectomy. While recurrence after complete resection is uncommon, metastasis to the contralateral distal ureter is exceptionally rare, with few cases reported. According to NCCN Guidelines for Kidney Cancer (v3.2025), post-nephrectomy surveillance will include history, physical examination, and laboratory assessment every six months for the first two years and annually thereafter, with abdominal and chest imaging every 6 - 12 months for at least five years. Extended surveillance beyond five years is recommended for patients with higher-risk histology or atypical metastatic behavior. The delayed metachronous recurrence in this patient underscores the importance of long-term follow-up in accordance with these guidelines, as well as thorough clinical history, interdisciplinary collaboration, and ongoing evaluation in the management of renal neoplasms.

## CRediT authorship contribution statement

**Kolos K. Nagy:** Writing – review & editing, Writing – original draft, Methodology, Conceptualization. **Caroline J. Cushman:** Writing – review & editing, Writing – original draft, Methodology, and Conceptualization. **Jason R. Jesudass:** Writing – review & editing. **Seshadri D. Thirumala:** Writing – review & editing, Supervision, Conceptualization. **Brian E. Nicholson:** Writing – review & editing, Supervision, Conceptualization. **Donald P. Quick:** Writing – review & editing, Supervision, Conceptualization.

## Disclosures

The authors have no disclosures, financial or otherwise.

## Consent

Written consent was obtained for this publication.
